# The 18-item Swedish version of Ryff’s psychological wellbeing scale: psychometric properties based on classical test theory and item response theory

**DOI:** 10.3389/fpsyg.2023.1208300

**Published:** 2023-10-03

**Authors:** Danilo Garcia, Maryam Kazemitabar, Mojtaba Habibi Asgarabad

**Affiliations:** ^1^Department of Behavioral Sciences and Learning, Linköping University, Linköping, Sweden; ^2^Centre for Ethics, Law and Mental Health (CELAM), University of Gothenburg, Gothenburg, Sweden; ^3^Promotion of Health and Innovation (PHI) Lab, International Network for Well-Being, Linköping, Sweden; ^4^Department of Psychology, University of Gothenburg, Gothenburg, Sweden; ^5^Department of Psychology, Lund University, Lund, Sweden; ^6^Yale School of Medicine, Yale University, New Haven, CT, United States; ^7^VA Connecticut Healthcare System, West Haven, CT, United States; ^8^Promotion of Health and Innovation (PHI) Lab, International Network for Well-Being, New Haven, CT, United States; ^9^Health Promotion Research Center, Iran University of Medical Sciences, Tehran, Iran; ^10^Department of Health Psychology, School of Behavioral Sciences and Mental Health (Tehran Institute of Psychiatry), Iran University of Medical Sciences, Tehran, Iran; ^11^Department of Psychology, Norwegian University of Science and Technology, Trondheim, Norway; ^12^Positive Youth Development Lab, Human Development and Family Sciences, Texas Tech University, Lubbock, TX, United States; ^13^Center of Excellence in Cognitive Neuropsychology, Institute for Cognitive and Brain Sciences, Shahid Beheshti University, Tehran, Iran

**Keywords:** classical test theory, item response theory, psychological wellbeing, psychometrics, wellbeing, health

## Abstract

**Background:**

Psychological wellbeing is conceptualized as the full engagement and optimal performance in existential challenges of life. Our understanding of psychological wellbeing is important for us humans to survive, adapt, and thrive during the challenges of the 21st century. Hence, the measurement of psychological wellbeing is one cornerstone for the identification and treatment of both mental illness and health promotion. In this context, Ryff operationalized psychological wellbeing as a six-dimensional model of human characteristics: self-acceptance, positive relations with others, environmental mastery, personal growth, autonomy, and purpose in life. Ryff’s Psychological Wellbeing Scale has been developed and translated into different versions. Here, we examine and describe the psychometric properties of the 18-item Swedish version of Ryff’s Psychological Wellbeing Scale using both Classical Test Theory (CTT) and Item Response Theory (IRT).

**Methods:**

The data used in the present study was earlier published elsewhere and consists of 768 participants (279 women and 489 men). In addition to the 18-item version of the scale, participants answered the Temporal Satisfaction with Life Scale, the Positive Affect Negative Affect Schedule, and the Background and Health Questionnaire. We examined, the 18-item version’s factor structure using different models and its relationship with subjective wellbeing, sociodemographic factors (e.g., education level, gender, age), lifestyle habits (i.e., smoking, frequency of doing exercise, and exercise intensity), and health issues (i.e., pain and sleeping problems). We also analyzed measurement invariance with regard to gender. Moreover, as an addition to the existing literature, we analyzed the properties of the 18 items using Graded Response Model (GRM).

**Results:**

Although the original six-factor structure showed a good fit, both CTT and IRT indicated that a five-factor model, without the purpose in life subscale, provided a better fit. The results supported the internal consistency and concurrent validity of the 18-item Swedish version. Moreover, invariance testing showed similar measurement precision by the scale across gender. Finally, we found several items, especially the purpose in life’s item “I live life one day at a time and do not really think about the future,” that might need revision or modification in order to improve measurement.

**Conclusion:**

A five-factor solution is a valid and reliable measure for the assessment of psychological wellbeing in the general Swedish population. With some modifications, the scale might achieve enough accuracy to measure the more appropriate and correct six-dimensional theoretical framework as detailed by Ryff. Fortunately, Ryff’s original version contains 20 items per subscale and should therefore act as a perfect pool of items in this endeavor.

## Background

In spite of the growing research on wellbeing (Barry et al., 2019), there is still a debate regarding its definition (for different viewpoints on wellbeing concepts, see Diener et al., 2018). At the conceptual level, researchers distinguish between hedonic and eudaimonic wellbeing. While hedonic wellbeing is often understood as subjective wellbeing or being satisfied with life, frequently experiencing positive affect, and infrequently experiencing negative affect (Bradburn, 1969; Diener et al., 1985; Pavot and Diener, 2008); eudaimonic wellbeing refers to psychological wellbeing or full engagement and optimal performance in existential challenges of life (Waterman, 1993; Ryan and Deci, 2001). In this context, Ryff (1989, 2014) operationalized psychological wellbeing as a six-dimensional model of human characteristics: self-acceptance, positive relations with others, environmental mastery, personal growth, autonomy, and purpose in life. Nevertheless, despite the debates over these definitions, researchers agree that both subjective and psychological wellbeing assess optimal psychological experience and functioning that are positively associated with each other (Myers and Diener, 1995; Ryan and Deci, 2001; Diener et al., 2018). Indeed, the Science of WellBeing (Cloninger, 2004, 2013) suggest that both flourishing and resilience is needed in order for us humans to survive, adapt, and thrive during the challenges of the 21st century. In this context, various measures have been developed for the assessment and operationalization of both subjective and psychological wellbeing. In this study, we focus on one of these measures, namely the 18-item version of Ryff’s Psychological Wellbeing Scale.

### Ryff’s psychological wellbeing scale

Research based on the outlook on wellbeing as the fulfillment or realization of one’s daimon or true nature throughout full engagement and optimal performance in the existential challenges of life (Ryan and Deci, 2001) has focused on what makes people healthier and enables them to adjust psychologically to changes in the environment (e.g., life style habits, such as being physically active and avoiding smoking). This approach goes back to Jahoda (1958), who tried to conceptualize “psychological health” based on developmental psychology theories that described positive mental health, including Jung’s account of individuation (Jung, 1933), Erikson’s psychosocial development (Erikson, 1959), Allport’s formulation of maturity (Allport, 1961), Roger’s depiction of a fully functioning person (Rogers, 1961), and Maslow’s conception of self-actualization (Maslow, 1968). On this basis, Ryff (1989, 1995, 2014) developed a multidimensional model that can be measured with an instrument consisting of 120 items—20 items per each psychological wellbeing dimension: self-acceptance (i.e., the knowledge, acceptance, and awareness of personal limitations), positive relations with others (i.e., the ability to create deep and meaningful connections with others), environmental mastery (i.e., the sense of control over one’s life situation), personal growth (i.e., the tendency to see life as a growing experience and as an opportunity to develop one’s talents and potential), autonomy (i.e., the sense of living in accordance with one’s own convictions and free will), and purpose in life (i.e., the tendency to perceive meaning, purpose, and direction in one’s own life). For administration purposes (e.g., time- and cost-effectiveness), Ryff has developed different versions containing 54 items, 42 items, 39 items, and 18 items.

The 18-item version comprises three of the original 20 items to assess each dimension (Ryff and Keyes, 1995). This shortened version’s subscales have correlation coefficients ranging from 0.70 to 0.89 with their corresponding subscales in the original 120-item version (Ryff and Keyes, 1995). Moreover, the study that first examined the 18-item version using Confirmatory Factor Analysis (CFA), supported the six-factor original multidimensional model of psychological wellbeing in a cohort of elderly Canadians (Clarke et al., 2001). Further studies using samples from different countries, such as Iranian (Khanjani et al., 2014), Portuguese (Fernandes et al., 2010), and Italian (Sirigatti et al., 2009) have also confirmed, using CFA, the goodness of fit of the 18-item version of Ryff’s Psychological Wellbeing Scale (see Table 1 for a compilation of some of these studies). Finally, Lindfors et al. (2006) showed that the internal consistency coefficients of one (of two) Swedish 18-item version were higher than those of the original 120-item version, suggesting adequacy of the shortened version in a sample of white-collar Swedish workers. Hence, most studies suggest that the 18-item version is relatively valid and reliable for the measurement of Ryff’s multidimensional model of psychological wellbeing.

**Table 1:** Studies validating the psychometric properties of the 18-item version of Ryff’s Psychological Well-Being Scale in different populations.

Country	Author	Participants	CFA	Reliability	
Iran	Khanjani et al. (2014)	*N* = 976 66.3% female and 33.7% male *M_age_* = 18.9 and *SD_age_* = 4.7	CFI (six-factor model) = 0.95	SA = 0.51 PR = 0.75 EM = 0.76	PG = 0.73 A = 0.72 PiL = 0.52
Taiwan	Li (2014)	*N* = 820 58% female and 42% male Age range: 31–95	CFI (Oblique six-factor model) = 0.98	SA = 0.73 PR = 0.71 EM = 0.75	PG = 0.74 A = 0.60 PiL = 0.73
Italy and Belarus	Sirigatti et al. (2013)	*N*_1_ = 619 83% female and 17% male 87% Age range: 15–18 13% Age range: 19 or older *N_2_* = 495 79% female and 21% male 70% Age range: 17–18 30% Age range: 19 and older	CFI 1 (six-factor correlate) = 0.98 CFI 2 (six-factor correlate) = 0.97	------	------
Portugal	Fernandes et al. (2010)	*N_1_* = 402 48.3% female and 51.7% male *M_age_* = 12.4 and *SD_age_* = 1.78 *N_2_* = 790 53.7% female and 46.3% male *M_age_* = 14.7 and *SD_age_* = 1.73	CFI 1 (six oblique factors) = 0.48 CFI 2 (six oblique factors) = 0.75	SA_1_ = 0.35 PR_1_ = 0.42 EM_1_ = 0.44 SA_2_ = 0.48 PR_2_ = 0.45 EM_2_ = 0.27	PG_1_ = 0.36 A_1_ = 0.50 PiL_1_ = 0.37 PG_2_ = 0.45 A_2_ = 0.36 PiL_2_ = 0.33
Italy	Sirigatti et al. (2009)	*N* = 602 83% female and 17% male 96% Age range: 13–18 4% Age range: 18 and older	CFI (six-factor model) = 0.84	SA = 0.82 PR = 0.81 EM = 0.31	PG = 0.78 A = 0.21 PiL = 0.81
Sweden	Lindfors et al. (2006)	*N* = 1,260 55% female and 45% male *M_age_* = 45.3 and *SD_age_* = 7.2	AGFI (six-factor correlate) = 0.95	SA = 0.70 PR = 0.65 EM = 0.71	PG = 0.66 A = 0.53 PiL = 0.24
Canada	Clarke et al. (2001)	*N* = 4,960 58% female and 42% male *M_age_* = 75.5 and *SD_age_* = 5.2	CFI (six-factor model) = 0.77	SA = 0.52 PR = 0.48 EM = 0.46	PG = 0.36 A = 0.37 PiL = 0.26
USA	Ryff and Keyes (1995)	*N* = 1,108 59% female and 41% male *M_age_* = 45.6 and *SD_age_* = 14.8	AGFI (six-factor model) = 0.89	SA = 0.52 PR = 0.56 EM = 0.49	PG = 0.40 A = 0.37 PiL = 0.33

PR, positive relations with others; EM, environmental mastery; SA, self-acceptance; A, autonomy; PG, personal growth; and PiL, purpose in life.

Nevertheless, there is only one study (Lindfors et al., 2006) confirming the psychometric properties of one of the Swedish 18-tem version. The other version, translated by Garcia (2006), has been used across many more studies and is yet to be validated. What is even more, to the best of our knowledge, all studies have exclusively used Classical Test Theory (CTT) to examine the psychometric properties of the 18-item version. Importantly, СTT has some limitations, such as the fact that item difficulty and item discrimination are group dependent, and therefore the results are dependent on the constitution of the sample (e.g., sample size, age, gender distribution, and *etcetera*). Additionally, scores obtained from CTT methods are completely test-dependent—since test difficulty directly influences the yielded test scores, this can influence the results. For instance, in their multi-group analyses, Sirigatti et al. (2013) showed that the factor structure of the 18-item version was the same across Italian and Belarusian samples. However, in a South African sample, the best solution was a two-factor model, in which all the positively worded items were clustered in the first factor and all the negatively worded items were grouped in the second factor (Henn et al., 2016). Furthermore, in their study, Hsu et al. (2017) showed that the discriminant validity of the 18-item version was questionable since five out of the six factors had considerable cross-loadings. Last but not least, the true-score model upon which much CTT is based on does not reflect participants’ responses to each specific item. As a result, it cannot be predicted how a participant will respond to a specific item (Hambleton and Jones, 1993).

More modern measurement theories, such as Item Response Theory (IRT) were developed to overcome the above-mentioned limitations. Specifically, IRT modeling provides a way of constructing psychological instruments and examining their measurement characteristics, including dimensionality of the instrument and the quality of response categories in Likert-type scales (Linacre, 2002). Thus, using IRT, researchers can improve measurement accuracy and reliability notably (An and Yung, 2014). Additionally, researchers can determine how many items are suitable to measure a specific construct with, and can therefore make significant reductions in assessment time and effort and, at the same time, increase the validity of the scale (e.g., Edelen and Reeve, 2007; Reise and Waller, 2009).

### The current study

Given the importance of fast and accurate assessment of psychological wellbeing as an indicator of mental health, many studies have used the 18-item version of Ryff’s Psychological Wellbeing Scale. However, the majority of these studies, if not all, have examined the psychometric properties of the scale using CTT (i.e., some types of factor analysis), showing mixed results. Therefore, we argue that there is a further need to study this specific 18-item version of Ryff’s Psychological WellBeing Scale and to replicate the results in different cultures (*cf.* Cohen, 1990, p. 1311). Thus, we aimed to examine and describe the psychometric properties of the 18-item Swedish version of Ryff’s Psychological WellBeing Scale translated by Garcia (2006); (see Table 2) using both Classical Test Theory (CTT) and Item Response Theory (IRT). This version has been used in many different studies across a wide range of participants (e.g., Garcia and Siddiqui, 2009; Garcia, 2011; Garcia et al., 2012, 2015, 2016a, 2017, 2018; Garcia and Moradi, 2013). We examined, for the first time, its factor structure using Structural Equation Modelling (SEM) and its relationship with subjective wellbeing, sociodemographic factors (e.g., education level, gender, age), lifestyle habits (i.e., smoking, frequency of doing exercise, and exercise intensity), and health issues (i.e., pain and sleeping problems). Moreover, as an addition to the existing literature, we analyzed the properties of the 18 items using Graded Response Model (GRM)—a specific method within IRT for measures that use Likert scales. In this endeavor, we use previously published data (e.g., Garcia et al., 2016c).

**Table 2:** The 18-item Swedish version of Ryff’s Psychological Well-Being Scale^1^.

No.	English	Swedish	Dimension (Scoring)
18	Maintaining close relationships has been difficult and frustrating for me.	Att upprätthålla nära relationer har varit svårt och frustrerande för mig.	PR (−)
17	The demands of everyday life often get me down.	Kraven i vardagslivet gör mig nedstämd.	EM (−)
16	In many ways, I feel disappointed about my achievements in life.	Jag känner mig på många sätt besviken med mina prestationer i livet.	SA (−)
15	I like most aspects of my personality.	Jag tycker om de flesta delarna av. min personlighet.	SA (+)
14	I have not experienced many warm and trusting relationships with others.	Jag har inte upplevt många varma och tillitsfulla relationer med andra.	PR (−)
13	I judge myself by what I think is important, not by what others think.	Jag dömer mig själv utifrån vad jag tycker är viktigt, inte efter det andra anser vara viktigt.	A (+)
12	In general, I feel I am in charge of the situation in which I live.	Generellt sett känner jag att jag har kontroll över min livssituation.	EM (+)
11	I am quite good at managing the responsibilities of my daily life.	Jag är bra på att hantera vardagens ansvar.	EM (+)
10	I tend to be influenced by people with strong opinions.	Jag har en tendens att påverkas av. människor med starka åsikter.	A (−)
9	I gave up trying to make big improvements or changes in my life a long time ago.	Jag har slutat försöka göra stora förbättringar eller förändringar i mitt liv för länge sedan.	PG (−)
8	I think it is important to have new experiences that challenge how you think about yourself and the world.	Jag tycker att det är viktigt att få nya erfarenheter som utmanar vad jag anser om mig själv och omvärlden.	PG (+)
7	I live life one day at a time and do not really think about the future.	Jag lever en dag i taget och tänker inte så mycket på framtiden.	PiL (−)
6	I have confidence in my own opinions, even if they are contrary to the general consensus.	Jag har tilltro till mina egna åsikter, även om de skiljer sig från hur de flesta andra tänker.	A (+)
5	För mig har livet varit en ständig process av. lärande, förändring och utveckling.	För mig har livet varit en ständig process av. lärande, förändring och utveckling.	PG (+)
4	For me, life has been a continuous process of learning, changing, and growth.	Vissa människor går genom livet utan några mål, men jag är inte en av. dem.	PiL (+)
3	People would describe me as a giving person, willing to share my time with others.	Andra skulle beskriva mig som en givmild person, villig att ta sig tid för andra.	PR (+)
2	I sometimes feel as if I’ve done all there is to do in life.	Ibland känner jag att jag har gjort allt som finns att göra i livet.	PiL (−)
1	When I look at the story of my life, I am pleased with how things have turned out.	När jag ser tillbaka på mitt liv är jag nöjd med hur saker och ting har blivit.	SA (+)

PR, positive relations with others; EM, environmental mastery; SA, self-acceptance; A, autonomy; PG, personal growth; and PiL, purpose in life. (−), negatively scored; (+), positively sored. The Likert scale scale is as follows [Swedish translations in brackets]: 1, Strongly Disagree [Stämmer inte alls]; 2, Disagree [Stämmer inte]; 3, Slightly Disagree [Stämmer delvis inte]; 4, Slighlty Agree [Stämmer delvis]; 5, Agree [Stämmer]; 6, Strongly Agree [Stämmer helt]. Instructions [Swedish translations in brackets]: For each statement below, indicate which answer best describes your present agreement or disagreement with each statement [Detta är en enkät som har att göra med hur du tänker i olika situationer. Läs igenom nedanstående påståenden och avgör hur mycket du håller med vart och ett enligt dina värderingar och erfarenheter]. ^1^Ryff’s Scales of Psychological Well-Being from Clarke et al. (2001). Translation to Swedish by Johanna Ekberg, Patricia Rosenberg & Danilo Garcia (Garcia, 2006). See Garcia and Siddiqui (2009).

## Methods

### Ethical statement

The present data was previously published open access (Garcia et al., 2016c). In the original study, after consulting with the Network for Empowerment and Well-Being’s Review Board we arrived at the conclusion that the design of the present study (e.g., all participants’ data were anonymous and will not be used for commercial or other non-scientific purposes) required only informed consent from the participants.

### Participants and procedure

In the original data collection (Garcia et al., 2016c), a total of 768 Swedish participants, including 279 women and 489 men, aged 13–76 years (*M* = 25.21; *SD* = 11.34) were selected using volunteer sampling. Participants reported a mean of 4.57 years of education (*SD* = 4.86) after ground school.

### Measures

#### Ryff’s psychological wellbeing scale (18 items)

The 18-item version of Ryff’s Psychological WellBeing Scale (Ryff and Keyes, 1995) is a self-report instrument that comprises 18 items measuring six dimensions of psychological wellbeing: autonomy, environmental mastery, self-acceptance, personal growth, positive relations with others, and purpose in life. The items are rated on a 6-point Likert scale, ranging from 1 (*strongly disagree*) to 6 (*strongly agree*). Therefore, the total score is in the range of 18–108, with higher scores representing greater wellbeing. The Swedish version of the scale used in this study was developed by Garcia (2006) and has been extensively used in several studies (e.g., Garcia et al., 2012, 2015; Garcia and Moradi, 2013). For the original study, the instrument was translated into Swedish by a bilingual Swedish teacher who was fluent in both English and Swedish. Then, a bilingual English teacher, fluent in Swedish, back-translated the instrument. A group of psychologists compared the back-translated version of the scale with the original version in order to check the quality and precision of translation. Any differences between these two versions were judged based on the consensus achieved by them, which led to the final 18-item Swedish version used here. Finally, to assess any misunderstanding and ambiguity in wording, this Swedish version was used in a pilot in a group of 30 participants, who were asked to rate readability and clarity of every single item on a six-point scale, ranging from 0 (*not understandable*) to 5 (*completely understandable*). For every item, “*completely understandable*” response option was endorsed by at least 95% of the respondents, and therefore, there was no need for item revision (Guillemin et al., 1993). Given that the subscales of the 18-item version show low to modest internal consistency, Garcia used and recommended that the total score is a better and more reliable measure of psychological wellbeing (Garcia and Siddiqui, 2009).

#### Temporal satisfaction with life scale

The Temporal Satisfaction with Life Scale (Pavot et al., 1998) is a 15-item measure that assesses the cognitive dimension of subjective wellbeing (i.e., an individual’s judgment of her/his global life satisfaction) in three areas, including past (e.g., “*If I had my past to live over, I would change nothing*”), present (e.g., “*I would change nothing about my current life*”), and future life satisfaction (e.g., “*There will be nothing that I will want to change about my future*”). The items are scored on a 7-point Likert scale ranging from 1 (*strongly disagree*) to 7 (*strongly agree*); therefore, the total score ranges from 15 to 105, with higher scores showing a higher level of life satisfaction. The Swedish version of the Temporal Satisfaction With Life Scale was also developed by Garcia and has shown satisfactory internal consistency (e.g., *Cronbach’s α* coefficients of 0.86 for present life satisfaction, 0.93 for past life satisfaction, and 0.88 for future life satisfaction) in past studies (Sailer et al., 2014).

#### Positive affect negative affect schedule

The Positive Affect Negative Affect Schedule (Watson et al., 1988) is a self-report measure that consists of 10 positive (e.g., proud, strong, and interested) and 10 negative affect items (e.g., nervous, afraid, and ashamed). This instrument is commonly used to assess the affective dimension of subjective wellbeing. Respondents are asked to use a 5-point Likert scale (1 = *very slightly or not at all* to 5 = *extremely*) to rate to what extent they have experienced the 20 feelings and emotions in the past 4 weeks. The Swedish version of the PANAS in this study has also been used in several studies (e.g., Archer et al., 2008; Garcia et al., 2016b) and has shown good reliability (*Cronbach’s α* coefficients of 0.84 for positive affect and 0.82 for negative affect) in different Swedish populations (e.g., Garcia et al., 2010).

#### Background and health questionnaire

The Background and Health Questionnaire (Rosén, 2002) is a self-report instrument used to collect background data and health-related information. The questionnaire consists of items regarding age, gender, education, lifestyle (e.g., frequency of doing exercise, sleep, smoking habits, and time spent watching TV), and health issues (e.g., pain and sleeping problems). For example: “*How often have you experienced sleep problems in the past year*” for which response options include “*Constantly*,” “*2–3 times a week*,” “*Once a week*,” “*Once a month*,” and “*Never*.” It is worth mentioning that this questionnaire has been widely used in Sweden as a reliable tool for gathering health-related information (e.g., Schütz et al., 2013, 2014).

### Data analysis strategy

Firstly, as it is recommended for ordinal Likert-type scales, the internal consistency of the subscales of the 18-item version was examined using the equivalent of *Cronbach’s alpha* coefficient, which is based on polychoric correlation matrix rather than the Pearson correlation matrix (Zumbo et al., 2007; Gadermann et al., 2012). Here, a correlation coefficient of 0.70 or higher was considered as an acceptable level of internal consistency of the items (see Cicchetti, 1994).

Secondly, we applied CFA using Weighted Least Square (WLS) to examine *a priori* models of the internal structure of the scale—this type of analysis provides less bias and more accurate results to ordinal Likert-type scales (Mindrila, 2010; Li, 2016). We used the following statistical tests and indices to assess the “goodness-of-fit” of the models (acceptable values in parenthesis): the Goodness-of-Fit Index (*GFI* > 0.95), the Adjusted Goodness-of-Fit Index (*AGFI* > 0.95), the Non-Normal Fit Index (*NNFI* > 0.95), the Comparative Fit Index (*CFI* > 0.95), the Root Mean Square Residual (*RMSR* < 0.05), The normal chi-square (*χ^2^/df* < 5), the Root Mean Square Error of Approximation (*RMSEA = 0.10: moderate fit, RMSEA = 0.08: reasonable fit, and RMSEA = 0.05: excellent fit.*), and its 90% confidence interval (Bentler and Bonett, 1980; MacCallum et al., 1996; Loehlin, 2003; Miles and Shevlin, 2007). We used multiple indices since they provide different information about the model fit, including the absolute fit, fit adjusting for model parsimony, and fit relative to a null model. Used together, these indices provide a more conservative and reliable evaluation (Maruyama, 1998). Moreover, the multivariate skewness in our data and the fit indices (except for SRMR) of all models were corrected using the *Satorra-Bentler scaled chi-square* test statistic (Hu et al., 1992; Bentler, 1995).

Third, we examined the fundamental assumptions for conducting graded response model (GRM) and ran the model. The GRM is a flexible and widely used IRT model for analyzing polytomous item responses, such as those found in Likert-type scales (Forero and Maydeu-Olivares, 2009). Unidimensionality of the subscales was measured by estimating internal consistency reliability and conducting one-factor CFA using LISREL 8.8 (Jöreskog and Sörbom, 2006). This statistical analysis allowed us to examine how well the items within each subscale collectively measured the same underlying construct. By conducting the one-factor CFA, we aimed to ensure that the items within each subscale were highly related and contributed to measuring the intended psychological wellbeing construct. The internal consistency analysis provided valuable insights into the degree of coherence and homogeneity among the items within each subscale. Moreover, local independence was evaluated by examining residual correlations among items in the one-factor model. The GRM model assumes that the item parameters (e.g., item difficulty, discrimination) are invariant across different groups or administrations of the test. This assumption ensures that the same underlying trait is measured consistently across different populations or time points (Zumbo, 1999). To measure invariance, we conducted Differential Item Functioning (DIF) across gender group. Finally, the GRM assumes that the probability of endorsing each response category for an item increases monotonically as the level of the latent trait increases (Embretson and Reise, 2013). In the context of wellbeing, this means that individuals with higher wellbeing levels are more likely to choose higher response categories on the items compared to those with lower wellbeing levels. Monotonicity was measured by conducting GRM and checking item fit. GRM was fit to the data and model, and item fit was determined using R 3.5.3. In addition, item scores were used to calibrate item “*difficulty*” on a logit scale with a midpoint of 0. Difficulty parameters were also inspected to determine whether items supported the comprehensive measurement of the underlying latent construct with minimal gaps and redundancy.

Fourth, the concurrent validity was evaluated by examining the correlations between scores in the different psychological wellbeing dimensions (i.e., self-acceptance, positive relations with others, environmental mastery, personal growth, autonomy, and purpose in life) and subjective wellbeing (i.e., temporal satisfaction with life, positive affect, and negative affect), demographic variables (i.e., gender, age, and education), lifestyle habits (i.e., smoking, frequency of doing exercise, and exercise intensity), and health issues (i.e., pain and sleeping problems).

Fifth, since the items of the 18-item version were ordinal and scored on a 6-point Likert scale, GRM (GRM; Samejima, 1968), conducted in MIRT 1.3 (Chalmers, 2012), was used as the appropriate IRT model (Edelen and Reeve, 2007; Ravens-Sieberer et al., 2008). The items that were reverse scored were coded so that higher scores represent greater psychological wellbeing. In GRM, each item has two types of parameters, including “*discrimination*” and “*threshold*.” Discrimination or “*slope*” parameter specified by “*ɑ*” shows the extent to which an item is related to, in this case, psychological wellbeing and how well an item discriminates between people with different levels of that latent trait. Items with higher discrimination parameters provide more information about psychological wellbeing. Generally, item discrimination values between 0.01–0.34 are considered “*very low*,” values between 0.34–0.64 are interpreted as “*low*,” values between 0.65–1.34 are “*moderate*,” values between 1.35–1.69 are “*high*,” and values >1.70 are considered “very high” (Baker, 2001). The other parameter, threshold, specified by “*b*,” refers to the point at which a respondent with a given level of psychological wellbeing has an equal probability (50:50) of responding above the threshold j (*j* = 1... *m_i_*, where *m_i_* + 1 = *K_i_* which is the number of response categories for item *i*). For each item, the number of threshold parameters is equal to the number of item response categories minus one (i.e., *K*-1).

Finally, DIF was used to examine the systematic errors (bias) due to gender (478 males vs. 276 females). Significant DIF values, evidenced by the logistic regression, indicate that one group of respondents has a higher or lower score on an item compared to another group after adjusting for the overall scores of the respondents. Having established the scale composition based on the results of the psychometric analyses, scale scores were calculated by averaging constituent items such that all scale scores ranged from 1 to 6, with higher scores indicating greater psychological wellbeing. Discriminative validity was also evaluated by testing expected gender- and grade-level differences (Langer et al., 2008). We also calculated between-group effect sizes (ES, d), which we interpreted as significant if they were higher than 0.20 (Cohen, 1988).

## Results

### Descriptive statistics

Table 3 shows descriptive statistics of the items in the 18-item Swedish version of Ryff’s Psychological Wellbeing Scale. As shown, all items were homogeneous and there was very little missing data, ranging from one missing response (i.e., 0.1% for items 1, 3, 9, 10, 11, 12, and 14) to seven missing responses (i.e., 0.9% for item 4). Given that missing data were at random and accounted for less than 5% (Pallant, 2005), we used listwise deletion without data imputation in order to handle the missing data. Of the 18 items, a ceiling effect was found only for three items (i.e., item 8: “I think it is important to have new experiences that challenge how you think about yourself and the world.,” item 9: “I gave up trying to make big improvements or changes in my life a long time ago.,” and item 14: “I have not experienced many warm and trusting relationships with others.”). Importantly, items 9 and 14 are negatively scored. Moreover, many of the respondents endorsed the last three response options of the six-point Likert scale, which might indicate the (in)frequency of these behaviors in the general Swedish population.

**Table 3:** Items’ response distribution, percentage missing, mean, standard deviation, skewness, kurtosis, corrected item-total correlations, and reliability of the 18-item Swedish version of Ryff’s Psychological Well-Being Scale.

Subscale^a^	Item No.	Response frequencies^b^ (%)						Missing (%)	*M*	SD	*r* ^cs^	*r* ^ct^	SK	KU	CAID	OTID	P.E	*T* value	Scale Reliability	Omega coefficient	Scale’s *M* (SD)
		1	2	3	4	5	6														
PR	18 (R)	4.8	9.5	15.0	13.9	32.3	24.5	–	4.33	1.47	0.43	0.59	–0.66	–0.60	0.77	4.50 (0.98)	0.85	31.14	0.524 (0.53)	0.60	13.49 (2.95)
	14 (R)	4.2	9.6	11.2	11.6	27.1	36.2	0.1	4.57	1.50	0.39	0.58	–0.84	–0.45	0.78	0.82	0.65	23.66			
	3	0.9	3.8	10.0	24.6	40.4	20.2	0.1	4.60	1.08	0.29	0.25	–0.77	0.42	0.78	0.82	0.47	14.64			
EM	17 (R)	7.0	15.5	22.4	17.1	24.2	13.5	0.3	3.77	1.48	0.48	0.51	–0.14	–1.02	0.77	4.23 (1.01)	0.90	42.08	0.685 (0.71)	0.71	12.69 (3.04)
	12	1.4	7.9	12.1	24.3	39.1	15.0	0.1	4.37	1.19	0.59	0.77	–0.69	−0.08	0.77	0.80	0.84	54.30			
	11	1.8	4.6	10.2	21.5	42.1	19.8	0.1	4.57	1.15	0.50	0.62	–0.92	0.60	0.77	0.81	0.78	44.81			
SA	16 (R)	3.6	9.9	14.2	17.2	28.0	26.4	0.7	4.36	1.44	0.63	0.72	–0.66	–0.60	0.76	4.25 (1.10)	0.90	69.14	0.767 (0.77)	0.78	12.75 (3.29)
	15	1.4	6.9	10.3	27.1	39.7	14.2	0.4	4.40	1.14	0.57	0.63	–0.75	0.22	0.77	0.80	0.78	35.24			
	1	5.9	10.8	14.7	27.4	29.7	11.5	0.1	3.99	1.36	0.59	0.73	–0.51	–0.49	0.76	0.80	0.76	38.92			
A	13	1.4	8.2	17.3	24.8	31.4	16.7	0.4	4.27	1.24	0.34	0.46	–0.43	–0.53	0.78	4.05 (0.89)	0.71	16.13	0.489 (0.49)	0.48	12.14 (2.66)
	10 (R)	4.2	16.7	27.6	22.7	21.1	7.7	0.1	3.63	1.30	0.14	0.36	0.013	–0.78	0.79	0.83	0.42	11.38			
	6	1.8	6.8	15.4	28.3	33.5	14.1	0.3	4.27	1.19	0.23	0.51	–0.53	−0.16	0.79	0.82	0.41	10.82			
PG	9 (R)	1.7	3.9	8.7	15.8	31.0	38.9	0.1	4.87	1.22	0.37	0.37	–1.10	0.67	0.78	4.80 (0.85)	0.76	26.19	0.552 (0.57)	0.58	14.39 (2.56)
	8	0.9	2.9	8.2	21.1	33.3	33.3	0.3	4.84	1.12	0.35	0.63	–0.91	0.50	0.78	0.82	0.67	24.37			
	5	1.6	3.5	7.8	24.1	36.5	26.6	–	4.70	1.13	0.33	0.53	–0.92	0.74	0.78	0.82	0.63	23.68			
PiL	7 (R)	2.5	9.2	19.5	22.5	31.3	14.7	0.3	4.15	1.29	–0.08	0.12	–0.41	–0.60	0.81	4.45 (0.78)	0.37	6.18	0.082 (0.11)	0.09	13.35 (2.34)
	4	5.1	10.2	18.4	21.9	25.4	18.2	0.9	4.08	1.43	0.37	0.09	–0.39	–0.72	0.78	0.82	0.08	2.59			
	2 (R)	1.0	3.6	5.2	10.0	27.6	52.5	–	5.17	1.14	0.14	0.28	–1.56	2.00	0.79	0.83	0.28	5.20			

M, mean; SD, standard deviation; P.E, Parameter estimation (factor loading) for six-factor oblique model; SK, Skewness; KU, kurtosis; CAID, Cronbach’s alpha if item deleted; OTID, ordinal theta if item deleted; r^cs^, corrected item-total correlation for subscales` items; r^ct^, corrected item-total correlation for total scale’s items. ^a^PR, positive relations with others; EM, environmental mastery; SA, self-acceptance; A, autonomy; PG, personal growth; and PiL, purpose in life. ^b^Response options were as follows: 1, strongly disagree; 2, moderately disagree; 3, slightly disagree; 4, slightly agree; 5, moderately agree; and 6, strongly agree. ^c^The coefficients outside and inside of parentheses show Cronbach’s alpha for each subscale and ordinal theta, respectively.

A preliminary analysis showed that for all six subscales, all items met the univariate outlier criteria [−3.00 > *Z _x_* > +3.00]. The decision about keeping or removing outliers was made based on a comparison between the original mean and a 5% trimmed mean. Since the presence or absence of outliers did not influence the main findings of the study, we included the outliers and used robust analyses for estimating relevant statistical parameters (Pallant, 2005; Tabachnick and Fidell, 2007). Testing the assumption of normality revealed a positive but non-substantial skewness in all sub-scales of the 18-item version—A value lower than 0.05 on the Kolmogorov–Smirnov test suggests that the normality assumption is violated.

### Scale’s internal consistency

As indicated in Table 3, the values of univariate skewness ranged from +0.01 for item 10 to −1.56 for item 2. As shown in Table 3, *Cronbach’s alpha* and *ordinal theta* coefficients for the total scale were in acceptable range, respectively. Furthermore, these statistics were 0.68 for environmental mastery, 0.76 for self-acceptance, 0.55 for personal growth, 0.52 for positive relations with others, and 0.48 for both autonomy, and 0.8 purpose in life (95% *CI*).

In addition, the intercorrelation matrix presented in Table 3 shows that almost all the items within five of the six subscales have a positive moderate relationship with each other—with values ranging from 0.14 to 0.63 (based on the corrected item-total correlation for subscale’s items) and from 0.25 to 0.77 (based on the corrected item-total correlation for total scale’s items). However, the items within the purpose in life subscale had non-significant to moderate relationships with each other, with coefficients ranging from −0.08 to 0.37 (based on the corrected item-total correlation for subscale’s items) and from 0.09 to 0.28 (based on the corrected item-total correlation for total scale’s items). Our results also indicated that removing the purpose in life item 7 (“I live life one day at a time and do not really think about the future”), slightly increases the internal consistency of the subscale. Finally, the intraclass correlation coefficients and mean of inter-item correlations were 0.79 (95% *CI* = 0.77 to 0.81) and 0.17 for the total scale; 0.53 (95% *CI* = 0.47 to 0.58) and 0.27 for the positive relations with others subscale; 0.69 (95% *CI* = 0.65 to 0.72) and 0.42 for the environmental mastery subscale; 0.77 (95% *CI* = 0.74 to 0.80) and 0.53 for the self-acceptance subscale; 0.48 (95% *CI* = 0.42 to 0.55) and 0.24 for the autonomy subscale; 0.56 (95% *CI* = 0.50 to 0.61) and 0.30 for the personal growth subscale; and 0.08 (95% *CI* = −0.035 to 0.19) and 0.03 (95% *CI* = −0.012 to 0.07) for the purpose in life subscale.

### Dimensionality, local independence assessment, and factor structure

**Figure 1:**
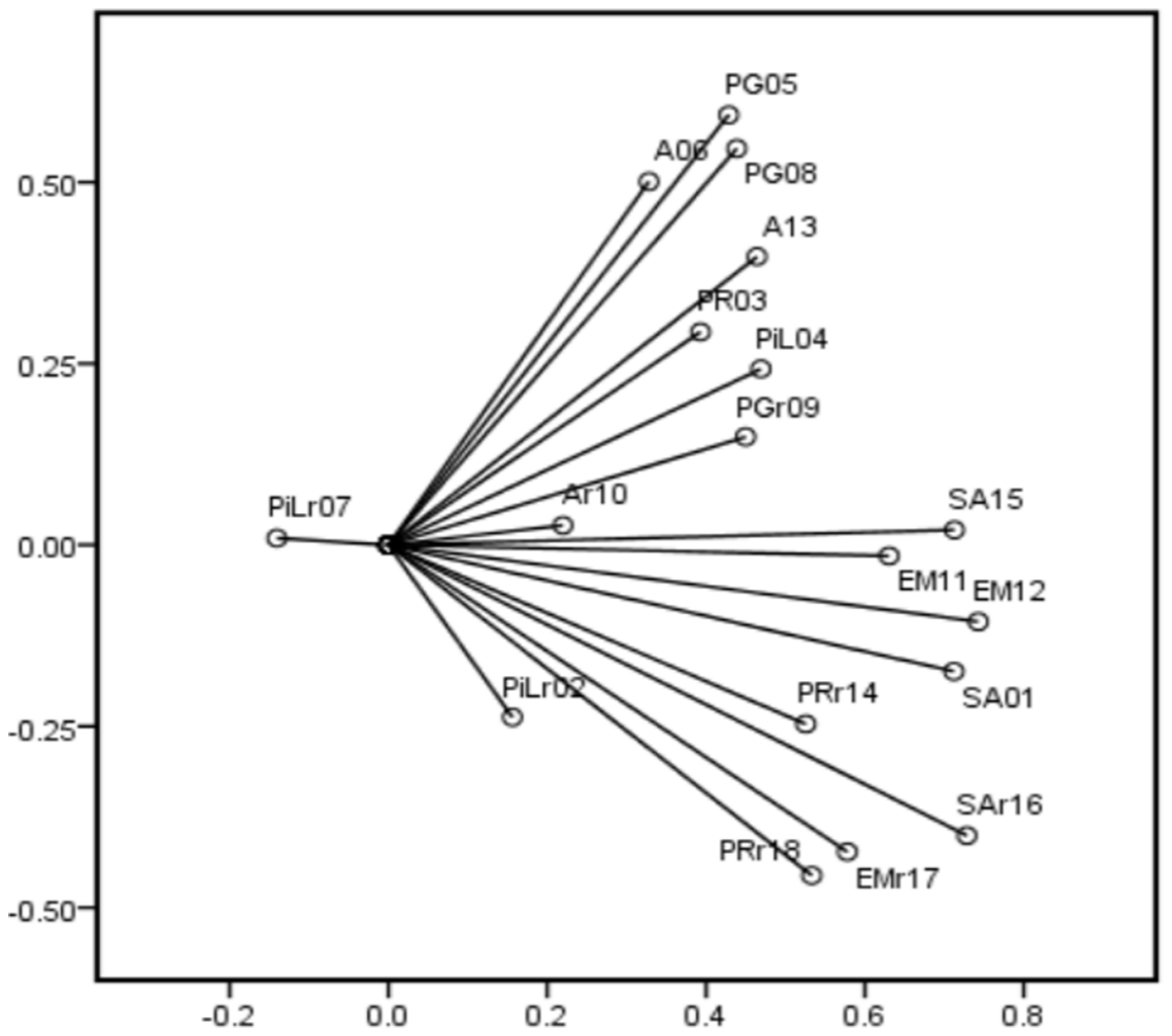
Loadings plot of dimensionality assessment of the 18-item Swedish version of Ryff’s Psychological Well-Being Scale by categorical principal component analysis.

Categorical principal component analysis (Mair and de Leeuw, 2010) was used to assess the dimensionality of the 18-item Swedish version of Ryff’s Psychological Wellbeing Scale. As the loadings plot in Figure 1 shows, the 18-item did not load efficiently on one general psychological wellbeing factor. Moreover, purpose in life item 7 (“I live life one day at a time and do not really think about the future”) pointed in a different direction compared to the other items within the subscale and the scale as a whole. Therefore, it is not surprising that the consistency of the items of this subscale was very low. Moreover, exploratory factor analysis based on polychoric correlations by parallel analysis (Revelle, 2017) indicated that there were five factors and four components. MAP test and Bic index also showed two and six factors, respectively. Hence, the 18-item Swedish version of Ryff’s Psychological Wellbeing Scale cannot be considered as a unidimensional scale with 18 items. The unidimensionality of the subscales was also assessed by one-factor CFAs using LISREL version 8.8 (Jöreskog and Sörbom, 2006). Local independence was also examined within each scale using paired residual correlations among items in the one-factor CFA model (Reeve et al., 2007). Given that the residual correlations were < 0.30 for all other item pairs within scales, local independence, an important premise for conducting IRT, was considered as confirmed.

As mentioned before, the internal consistency of all subscales of the 18-item version, except for purpose in life (*Cronbach’s alpha* = 0.08), was satisfactory. According to the Root Mean Square Error of Approximation (RMSEA) and the Comparative Fit Index (CFI), the one-factor CFA model fits our data well for five out of the six subscales. Specifically, as with its poor internal consistency, the purpose in life subscale was also a poor fit for the one-factor CFA model [*RMSEA* = 0.09, *CFI* = 0.82, see Hu and Bentler, 1999 who recommend an acceptable model fit of *RMSEA* ≤ 0.06 and *CFI* ≥ 0.95]. Moreover, despite the removal of items, unidimensionality for the purpose in life subscale was not confirmed.

To further examine the six-factor structure of the 18-item version, as suggested by Ryff (1995), the CFA was conducted using LISREL 8.8 (Jöreskog and Sörbom, 2006), and the goodness of fit was tested for eight models: Model 1 (*M*_1_) examined a one-factor model in which all the 18 items were loaded on a single psychological wellbeing factor; Model 2 (*M*_2_) consisted of a two-factor orthogonal model in which all the positively worded items were clustered in the first factor and all the negatively worded items were grouped in the second factor (Henn et al., 2016); Model 3 (*M*_3_) consisted of a six-factor orthogonal model; Model 4 (M_4_) examined a six-factor oblique model resembling the exploratory factor analysis conducted by Ryff (1995); Model 5 (*M*_5_) tested a six-factor first-order model and one-factor second-order that loaded by all six first-order factors; Model 6 (*M*_6_) evaluated a six-factor and four-factor second-order model that loaded by a four-factor model based on the four most highly correlated dimensions: environmental mastery, personal growth, purpose in life, and self-acceptance (Ryff and Singer, 2006; Springer et al., 2006); and finally, model 7 (*M*_7_) assessed a five-factor first-order oblique model after removing the purpose in life subscale. The oblique model was used because we expected the factors to be correlated with each other based on a theoretical standpoint. Moreover, the variance of each factor was set to 1.0 for all models.

**Figure 2:**
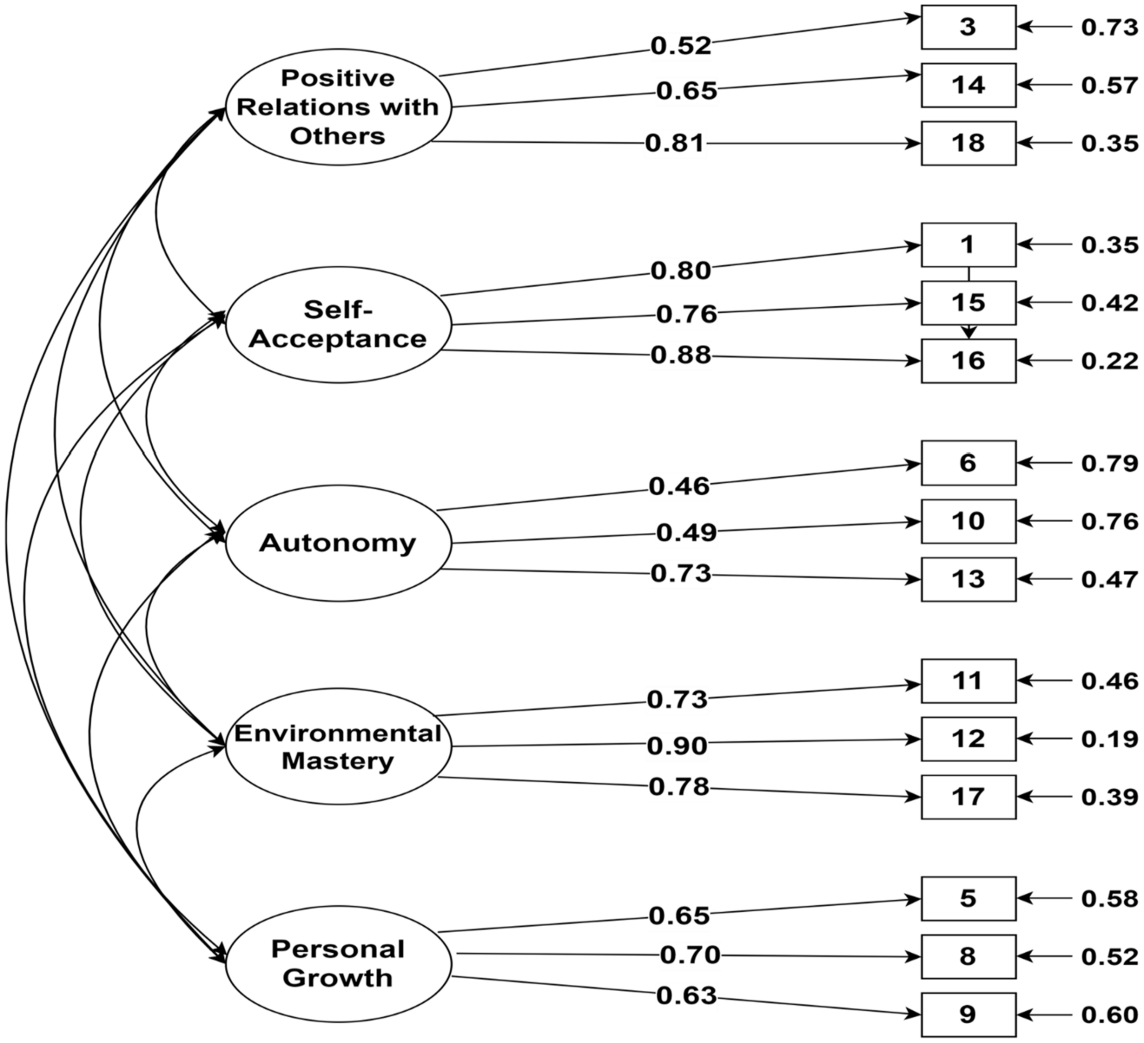
Path diagram of the five-factor oblique model for the 18-item Swedish version of Ryff’s Psychological Well-Being Scale.

As indicated in Table 4, the fit indices for none of the models met most of the specified fit criteria (i.e., *RMSEA* < 0.05 and *χ^2^/df* < 5). Although the six-factor oblique model, as the prior and theory-derived model (M_4_), provided a better fit, it was not completely satisfactory (*χ^2^/df* = 5.55; *CFI* = 0.89; *NNFI* = 0.85; and *RMSEA* = 0.077; 90% *CI* = 0.071–0.083). However, after removing the purpose in life scale, a five-factor first-order oblique model (M_7_) provided a better fit (*χ^2^/df* = 4.35; *CFI* = 0.92; *NNFI* = 0.90; and *RMSEA* = 0.066; 90% *CI* = 0.059–0.073), but not perfect goodness-of-fit. Path diagram for model 7 is shown in Figure 2. Then, the parsimonious principle (Bollen, 1989) was used to compare the fit indices of the *M*_1–7_ as nested models with those of the M_0_ as the baseline/null model. The comparison between the *M*_4_ with the *M*_2_ (*Δχ^2^* = 300.14, *Δdf* = 14, *p* < 0.001) and *M*_5_ (*Δχ^2^* = 69.72, *Δdf* = 9, *p* < 0.001) as competitive models, indicated that the six-factor oblique model (*M*_4_) was the optimal model. However, the comparison of the *M*_4_ with the *M*_7_ (*Δχ^2^* = 318, *Δdf* = 40, *p* < 0.0001) demonstrated that the five-factor first-order oblique model (*M*_7_) is the final parsimonious model. Factor loadings for the five-factor oblique model ranged from 0.46 to 0.90, and each item showed adequate factor loading on the related factor.

**Table 4:** Goodness-of-fit indices for confirmatory factor analysis of the 18-item Swedish version of Ryff’s Psychological Well-Being Scales.

Model	NNFI	AGFA	GFI	ECVI	CAIC	RMSEA	CFI	S-B χ^2^(df)	χ^2^/df	ΔS-B χ^2^(Δdf)
*M*_0_	0.72	0.91	0.93	1.63	1538.64	0.106 (0.10–0.11)	0.78	1148.81 (120)	9.57	
*M*_1_	0.79	0.93	0.94	1.42	1293.36	0.092 (0.087–0.097)	0.81	1018.18 (135)	7.54	130.63 (15)^**^
*M*_2_	0.80	0.93	0.94	1.35	1249.72	0.090 (0.084–0.095)	0.83	966.90 (134)	7.21	181.91 (14)^**^
*M*_3_	0.39	0.82	0.86	3.98	3256.63	0.16 (0.15–0.16)	0.40	2684.58 (135)	19.88	1535.77 (15)^**^
*M*_4_	0.85	0.95	0.96	1.00	1056.59	0.077 (0.071–0.083)	0.89	666.76 (120)	5.55	482.05^**^
*M*_5_	0.52	0.83	0.87	0.87	879.86	0.067 (0.062–0.073)	0.58	597.04 (129)	4.62	551.77 (9)^**^
*M*_6_	0.23	0.76	0.81	1.24	1150.13	0.085 (0.079–0.090)	0.32	882.60 (136)	6.48	266.21 (16)^**^
*M*_7_	0.90	0.97	0.98	0.559	654.51	0.066 (0.059–0.073)	0.92	348.76 (80)	4.35	800.05 (40)^**^

*M*_0_, model with random item-factor associations, *M*_1_, general one-factor for 18 items; *M*_2_, two-factor orthogonal model; *M*_3_, six-factor orthogonal model; *M*_4_, six-factor oblique model; *M*_5_, six-factor first-order and second order model; *M*_6_, a six-factor first-order model and four-factor second-order that loaded by a four-factor model based on the four most highly correlated dimensions: environmental mastery, personal growth, purpose in life and self-acceptance; *M*_7_, five-factor first-order oblique model after removing the purpose in life subscale. **p < 0.001.

After that, in order to examine whether or not the factor structure of the 18-item Swedish version of Ryff’s Psychological Wellbeing Scale was equivalent across gender, multiple-group CFA (Meredith, 1993) was conducted using R package lavaan (Rosseel, 2012). The basic definitions of the five levels of factorial invariance were: configural invariance (the pattern of fixed and free parameters), weak factorial invariance (corresponding factor loadings), strong factorial invariance (corresponding indicator means), strict factorial invariance (corresponding indicator residuals), and finally latent variances and covariance (Byrne et al., 1989; Cheung and Rensvold, 2002). In the case of configural invariance, χ^2^, RMSEA, CFI, NNFI, and other fit indices were used to examine whether or not the combined models have good model fit. Furthermore, for metric, scalar, residual, and latent variances and covariance, the RMSEA values and RMSEA confidence intervals of the hierarchical (nested) models were compared. For example, in the case of comparison of the weak and strong factorial invariance models, if the RMSEA values fall within one another’s confidence intervals, this shows strong factorial invariance. Then, the changes in the CFI of hierarchical (nested) models were examined. Also, the change in CFI for the weak and strong factorial invariance models was assessed. If the change is less than 0.01, this shows strong factorial invariance (Cheung and Rensvold, 2002). *Satorra-Bentler χ^2^* statistics was used to compare all constraint models presented in Table 5 with the staring model (Mo_1_), in which no constraint was imposed on the estimation of parameters, except for the mean of latent variables that was constrained to zero. Given that changes in the model fit index were minimal, metric invariance was established for the five-factor oblique model across gender. As indicated in Table 5, the hypothesized factor structure of the 18-item version (i.e., five-factor oblique model) fits the data well across gender, showing that the same construct is being measured across gender. That is, it shows equal form (i.e., the number of factors and the pattern of factor-indicator relationships are the same), equal factor loadings, equal thresholds (i.e., when observed scores are regressed on each factor, the thresholds are equal), and equal residual variances across women and men.

**Table 5:** Multiple invariance analysis for confirmatory factor analysis of the 18-item Swedish version of Ryff’s Psychological Well-Being Scale across gender.

Model	χ2	S-B χ2	df	*P*-value	AIC	BIC	CFI	TLI	RMSEA	SRMR	Base model	ΔS-B χ2	Δdf	Pr (> χ2)	ΔCFI	ΔRMSEA	ΔRMR	ΔTLI	ΔAIC	ΔBIC
Mo_1_	611.523	695.12	160	0.0001	35,684	36,195	0.831	0.779	0.091 (0.084–0.099)	0.073	–	–	–	–	–	–	–	–	–	–
Mo_2_	615.883	712.41	170	0.0001	35,682	36,146	0.830	0.791	0.089 (0.081–0.096)	0.077	Mo_1_	11.703	10	0.305	–0.001	–0.003	0.004	0.012	–2.713	–49.151
Mo_3_	623.191	715.55	180	0.0001	35,665	36,083	0.833	0.805	0.086 (0.079–0.093)	0.077	Mo_2_	3.128	10	0.978	0.002	–0.003	0.000	0.014	–16.862	–63.300
Mo_4_	633.728	737.69	195	0.0001	35,657	36,005	0.832	0.819	0.083 (0.075–0.090)	0.078	Mo_3_	16.350	15	0.359	–0.001	–0.003	0.001	0.014	–7.859	–77.515
Mo_5_	640.813	741.26	200	0.0001	35,650	35,976	0.832	0.824	0.081 (0.074–0.089)	0.081	Mo_4_	4.095	5	0.535	0.000	–0.001	0.003	0.005	–6.428	–29.647

fl, factor loadings; inter, item intercepts; *F*mean, mean of latent variable; var., variance of latent variable; cov, covariance of latent variable; inv, invariances; uniq, strict model invariances also known as residual or invariant uniqueness. Mo_1_ → inv = none; free = fl + inter + uniq + var. + cov. Mo_2_ → inv = fl; free = inter + uniq + var. + cov. Mo_3_ → inv = fl + inter; free = Fmean + uniq + var. + cov. Mo_4_ → inv = fl + inter + uniq; free = inter + Fmean + var. + cov. Mo_5_ → inv = fl + inter + uniq + var. + cov; free = inter + Fmean. Δ = differences between parameters of two models. S-B χ^2^ = significant χ^2^ change indicates non-invariance of the model that hierarchically was compared with the previously ordered model; modification index can be used to identify which estimated parameters are non-invariant across gender.

### Concurrent validity

Table 6 presents the Pearson correlation coefficients between the total psychological wellbeing score and each subscale’s score with the three subjective wellbeing components, demographics, lifestyle habits, and health. Almost all of the psychological wellbeing subscales were significantly associated with each other, with correlation coefficients ranging from 0.1 to 0.68. However, no significant relationship was found between purpose in life and autonomy (*r* = −0.02, ns). Moreover, as Table 6 shows, psychological wellbeing was positively associated with both temporal satisfaction with life (*r* = 0.65, *p* < 0.01) and positive affect (*r* = 0.58, *p* < 0.01), but negatively associated with negative affect (*r* = −0.52, *p* < 0.01). With regard to sociodemographic factors, lifestyle habits and health issues, psychological wellbeing was negatively correlated with a high frequency of feeling pain (*r* = −0.24, *p* < 0.01) and sleeping problems (*r* = −0.22, *p* < 0.01); it was positively associated with age (*r* = 0.13, *p* < 0.01), exercise frequency (*r* = 0.17, *p* < 0.01), and exercise intensity (*r* = 0.17, *p* < 0.01), but negative association with smoking (*r* = −0.14, *p* < 0.01), and it was not significantly associated with gender (*r* = 0.05), or education (*r* = 0.06).

**Table 6:** The correlation matrix between psychological well-being and subjective well-being, demographic variables, lifestyle habits, and health.

		1	2	3	4	5	6	7
Psychological Well-Being	Positive Relations with Others (1)	–						
	Environmental Mastery (2)	0.40^**^	–					
	Self–Acceptance (3)	0.50^**^	0.68^**^	–				
	Autonomy (4)	0.09^**^	0.28^**^	0.26^**^	–			
	Personal Growth (5)	0.26^**^	0.26^**^	0.34^**^	0.24^**^	–		
	Purpose in Life (6)	0.18^**^	0.17^**^	0.14^**^	– 0.02	0.35^**^	–	
	Psychological Well-Being (7)	0.66^**^	0.76^**^	0.81^**^	0.48^**^	0.62^**^	0.44^**^	–
Subjective Well-Being	Temporal Satisfaction with Life (8)	0.46^**^	0.61^**^	0.72^**^	0.19^**^	0.23^**^	0.12^**^	0.65^**^
	Positive Affect (9)	0.33^**^	0.53^**^	0.50^**^	0.24^**^	0.32^**^	0.22^**^	0.58^**^
	Negative Affect (10)	–0.32^**^	–0.54^**^	–0.50^**^	–0.28^**^	–0.20^**^	–0.05	–0.52^**^
Socio-demographics	Gender (11)	0.04	0.05	0.05	0.03	0.01	−0.01	0.05
	Age (12)	0.10^**^	0.20^**^	0.09^*^	0.07	0.03	−0.03	0.13^**^
	Education (13)	0.06	−0.05	0.06	0.002	0.11^**^	0.04	0.06
Life Style Habits	Exercise Frequency (14)	0.10^**^	0.20^**^	0.16^**^	0.02	0.02	0.12^**^	0.17^**^
	Exercise Intensity (15)	0.10^**^	0.18^**^	0.12^**^	0.06	0.08^*^	0.13^**^	0.17^**^
	Smoking (16)	−0.09^*^	−0.14^**^	–0.09^*^	−0.03	–0.09^*^	–0.10^**^	−0.14^**^
Health	Pain (17)	–0.14^**^	–0.33^**^	–0.25^**^	–0.05	–0.10 ^**^	0.05	–0.24^**^
	Sleeping Problems (18)	–0.12^**^	–0.29^**^	–0.27^**^	0.01	–0.04	–0.09^*^	–0.22^**^

^**^P < 0.01; *P < 0.05.

### Graded response model and differential item functioning

Table 7 demonstrates the GRM-IRT parameter estimates of the 18 items in the 18-item Swedish version of Ryff’s Psychological Wellbeing Scale. Discrimination or slope parameter (a) is proportional to the steep of the item characteristic curve. Compared to items with less steep slopes, those with steeper slopes are more useful for separating respondents into different levels of psychological wellbeing. Theoretically, a parameter can vary from -∞ to +∞; but typically range from about 0.5 to +2.5 (Edelen and Reeve, 2007). Items with negative, zero, and near-zero slopes show that the probability of answering the item with higher scores decreases for individuals with high levels of psychological wellbeing, which is counterintuitive and therefore indicates that such items should be modified or deleted (Baker, 2001). As indicated in Table 7, there is only one negative slope value, the purpose in life item 7 (“I live life one day at a time and do not really think about the future”; −0.218), which therefore needs to be revised in future studies. Moreover, the self-acceptance item 16 (“In many ways, I feel disappointed about my achievements in life”; 2.843), had the highest discrimination estimation, while the purpose in life item 2 (“I sometimes feel as if I’ve done all there is to do in life”; 0.185) had the lowest. In addition, the environmental mastery item 12 (“In general, I feel I am in charge of the situation in which I live”) and the self-acceptance items 16, 1, and 15 (“In many ways, I feel disappointed about my achievements in life”; “When I look at the story of my life, I am pleased with how things have turned out.”; and “I like most aspects of my personality”) had the highest discrimination estimations. In contrast, the lowest slope parameters belonged to the purpose in life items 7 and 2 (“I live life one day at a time and do not really think about the future”; “I sometimes feel as if I’ve done all there is to do in life”) and to the autonomy item 10 (“I tend to be influenced by people with strong opinions”). See Table 7 for the details.

**Table 7:** Graded Response Model IRT parameter estimates of the 18-item Swedish version of Ryff’s Psychological Well-Being Scale.

		Item parameter estimates						Fit Index			Gender DIF^b^		Gender observed means^c^	
Item no.	Subscale	*a*	*d1*	*d2*	*d3*	*d4*	*d5*	S-X^2^	df	RMSEA	χ^2^	adj_pvals^†^	Male	Female
18 (R)	PR	1.115	3.473	2.148	1.104	0.374	−1.369	33.94*	17	0.036	4.489	0.481	4.38	4.25
			0.188	0.123	0.098	0.090	0.103							
14 (R)		0.999	3.518	2.148	1.330	0.689	−0.659	20.96	20	0.008	8.409	0.405	4.50	4.60
			0.193	0.121	0.099	0.089	0.089							
3		0.594	4.878	3.184	1.873	0.461	−1.482	41.36*	19	0.039	4.842	0.481	4.61	4.61
			0.383	0.176	0.109	0.080	0.097							
17 (R)	EM	1.401	3.267	1.643	0.287	−0.637	−2.386	23.05	18	0.019	1.520	0.911	3.82	3.68
			0.173	0.114	0.095	0.097	0.136							
12		2.039	6.342	3.750	2.167	0.257	−2.909	9.54	13	0.001	0.196	0.310	4.41	4.31
			0.398	0.216	0.158	0.123	0.183							
11		1.320	4.919	3.474	2.130	0.611	−1.899	34.62*	16	0.039	10.214	0.208	4.62	4.51
			0.301	0.187	0.132	0.101	0.125							
16 (R)	SA	2.843	5.035	3.058	1.572	0.327	−1.724	7.79	14	0.001	1.187	0.964	4.43	4.26
			0.284	0.185	0.139	0.121	0.142							
15		2.172	5.886	3.608	2.218	0.225	−2.766	34.97*	15	0.042	2.943	0.964	4.43	4.35
			0.368	0.201	0.148	0.113	0.166							
1		2.518	4.147	2.525	1.251	−0.538	−3.106	21.97	14	0.027	0.979	0.964	4.04	3.92
			0.224	0.156	0.124	0.115	0.178							
13	A	0.774	4.555	2.482	1.093	−0.113	−1.836	16.85	17	0.000	3.556	0.704	4.29	4.22
			0.311	0.134	0.092	0.082	0.110							
10 (R)		0.286	3.169	1.354	0.057	−0.923	−2.524	25.02	19	0.020	2.972	0.704	3.65	3.60
			0.181	0.090	0.073	0.081	0.137							
6		0.429	4.098	2.456	1.190	−0.119	−1.906	34.43*	17	0.037	4.880	0.704	4.28	4.25
			0.272	0.133	0.088	0.076	0.109							
9 (R)	PG	0.780	4.349	3.079	1.979	0.956	−0.508	22.557	19	0.016	5.716	0.335	4.90	4.83
			0.286	0.167	0.114	0.089	0.083							
8		0.609	4.923	3.443	2.119	0.743	−0.776	18.859	14	0.021	8.438	0.201	4.81	4.87
			0.384	0.196	0.118	0.084	0.085							
5		0.597	4.349	3.110	2.048	0.580	−1.11	11.561	16	0.001	10.009	0.201	4.71	4.69
			0.295	0.171	0.115	0.082	0.09							
7 (R)	PiL	−0.218	3.696	2.041	0.79	−0.159	−1.773	22.33	20	0.012	3.910	0.743	4.08	4.28
			0.233	0.113	0.070	0.073	0.103							
4		0.737	3.226	1.938	0.739	−0.281	−1.675	33.52*	21	0.028	2.735	0.743	4.09	4.03
			0.175	0.112	0.086	0.082	0.104							
2 (R)		0.185	4.570	3.024	2.217	1.396	0.097	25.81	21	0.017	2.723	0.743	5.23	5.07
			0.356	0.171	0.121	0.091	0.073							

PR, positive relations with others; EM, environmental mastery; SA, self-acceptance; A, autonomy; PG, personal growth; and PiL, purpose in life. ^a^Item discrimination parameter estimates; *b1, b2, b3, b4*, and *b5*, item threshold parameter estimates; (R), reverse coded. ^b^Gender Differential Item Functioning (DIF) was tested using the likelihood ratio-based significance test under the IRT framework (IRT-LR) in an iterative purification procedure for identifying DIF free anchor set. ^c^Gender observed means are reported on a six-response category scale. ^*^p < 0.05; ^†^*p* < Benjamini–Hochberg adjusted overall alpha level of 0.05.

Table 7 also shows the intercept parameters (*d*) for the 18-item Swedish version of Ryff’s Psychological Wellbeing Scale. This parameter is the theta value that should have a probability of 0.5 for adjacent categories and represents the threshold required to move from point 1–2 (*d1*) in the Likert scale, point 2–3 (*d2*), and so on. The fit indices in Table 7 indicated low fit with the model for the positive relations with others items 18 and 3 (“Maintaining close relationships has been difficult and frustrating for me”; “People would describe me as a giving person, willing to share my time with others”); for the environmental mastery item 11 (“I am quite good at managing the responsibilities of my daily life”); for the self-acceptance item 15 (“I like most aspects of my personality”); for the autonomy item 6 (“I have confidence in my own opinions, even if they are contrary to the general consensus”); and for the purpose in life item 4 (“Some people wander aimlessly through life, but I am not one of them”). However, the model is considered poorly fitted.

Moreover, Table 7 shows DIF examined by the likelihood ratio-based significance test under the IRT framework (IRT-LR; Thissen et al., 1986). First, as recommended, we created a baseline model in which all items have the *a* and *d* parameters constrained across groups. Furthermore, the model contained freely estimated latent mean and variance in all but one group, namely the “*reference*” group. Such a model fixes the metric of the groups so that item parameter estimates do not contain latent distribution characteristics. The results are anchor items that are DIF-free and items that are suspected of DIF. Next, anchor items were used to study items that show DIF based on freeing *a* and *d* parameters, respectively. In this study, since all chi-square values were non-significant (*p* > 0.05), each subscale was analyzed separately. For each subscale, none of its three items showed DIF; thus, we regarded them as anchor items, and there was no need to proceed with the analysis. Also, as indicated in Table 7, the means of items for males and females were close to each other, confirming the results of the DIF analysis.

**Figure 3:**
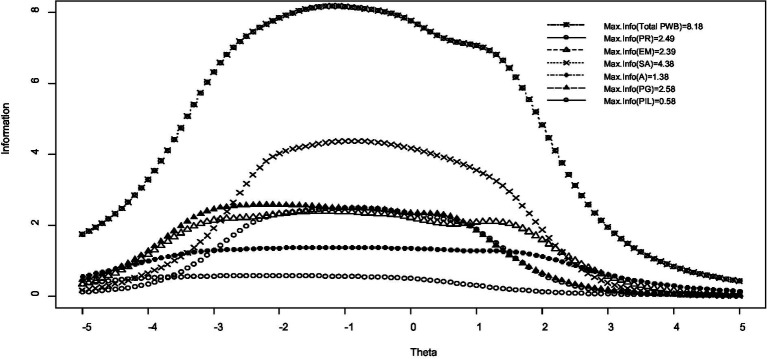
Information curves for the total scale and the subscales of the 18-item Swedish version of Ryff’s Psychological Well-Being Scale. PWB, psychological well-being total score; PR, positive relations with others; EM, environmental mastery; SA, self-acceptance; A, autonomy; PG, personal growth; and PiL, purpose in life.

Furthermore, Figure 3 shows the scale’s and subscales’ test information. The minimum value of test information is 0, which indicates that the test provides no information about the latent trait. In this case, the test cannot distinguish between individuals with different trait levels effectively. The maximum value of test information is unbounded in theory. However, in practice, the maximum value is influenced by the number of items, their psychometric properties, and the range of latent trait levels covered by the test. As the number of items increases, the maximum value of test information also increases, allowing the test to provide more precise estimates of individuals’ trait levels (Baker, 2001; Embretson and Reise, 2013).

**Figure 4:**
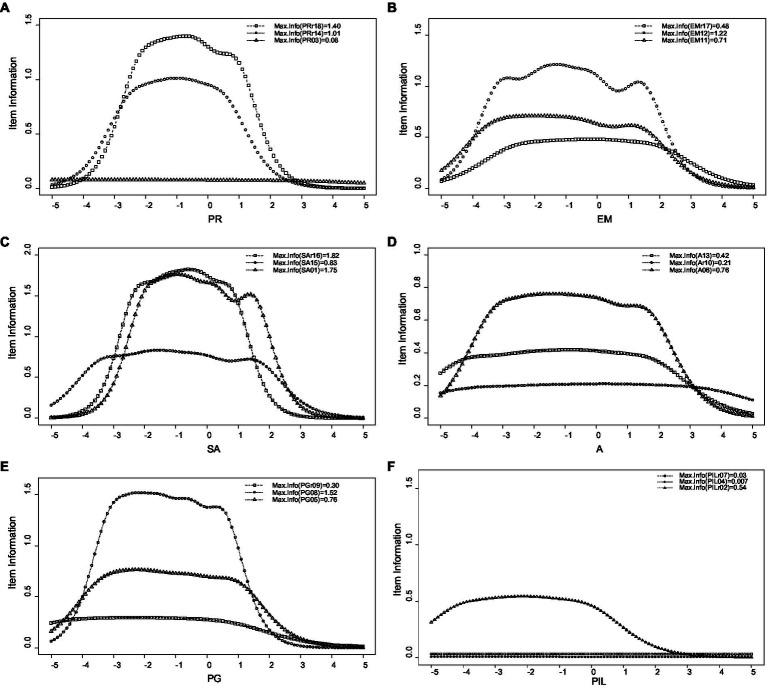
Item information of the 18-item Swedish version of Ryff’s Psychological Well-Being Scale’s subscales: positive relations with others **(A)**, environmental mastery **(B)**, self-acceptance **(C)**, autonomy **(D)**, personal growth **(E)**, and purpose in life **(F)**. PR, positive relations with others; EM, environmental mastery; SA, self-acceptance; A, autonomy; PG, personal growth; and PiL, purpose in life.

**Figure 5:**
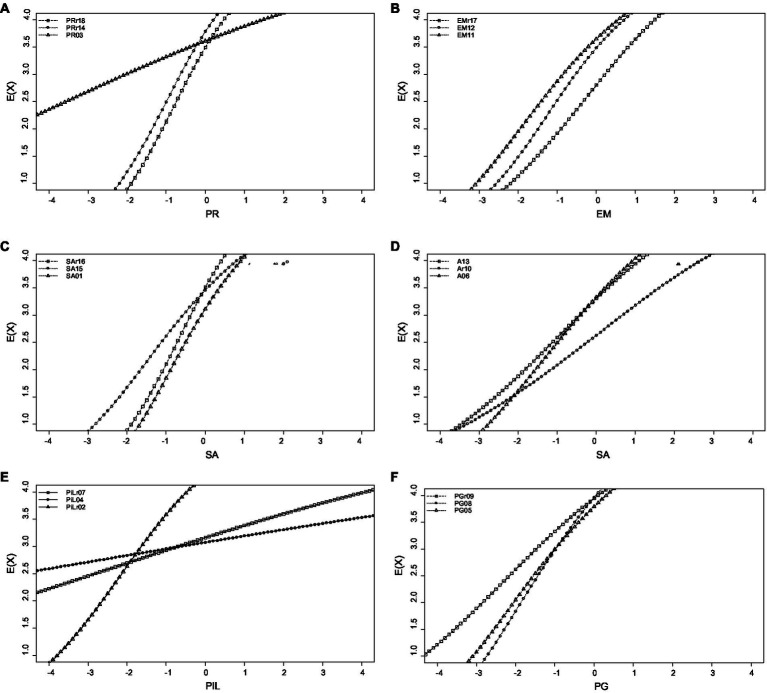
Expected item score of the 18-item Swedish version of Ryff’s Psychological Well-Being Scale’s subscales: positive relations with others **(A)**, environmental mastery **(B)**, self-acceptance **(C)**, autonomy **(D)**, personal growth **(E)**, and purpose in life **(F)**. PR, positive relations with others; EM, environmental mastery; SA, self-acceptance; A, autonomy; PG, personal growth; and PiL, purpose in life.

As indicated, except for the total psychological wellbeing score and the self-acceptance subscale, the subscales’ information is very low—especially for the purpose in life scale. Therefore, it is necessary to revise these subscales. Furthermore, with regard to the total psychological wellbeing score, only respondents with theta values between −2 to 1 were measured accurately. Additionally, as shown in Figure 4, the following items had low information and need revision: the positive relations with others item 3 (“People would describe me as a giving person, willing to share my time with others”); the environmental mastery items 17 and 11 (“The demands of everyday life often get me down”; “I am quite good at managing the responsibilities of my daily life”); the self-acceptance item 15 (“I like most aspects of my personality”); the autonomy items 10 and 13 (“I tend to be influenced by people with strong opinions”; “I judge myself by what I think is important, not by what others think”); the personal growth items 5 and 9 (“For me, life has been a continuous process of learning, changing, and growth”; “I gave up trying to make big improvements or changes in my life a long time ago”); and the purpose in life subscale items 4 and 7 (“Some people wander aimlessly through life, but I am not one of them”; “I live life one day at a time and do not really think about the future”). Finally, the expected item score for the subscales of the 18-item Swedish version of Ryff’s Psychological Wellbeing Scale is presented in Figure 5. As indicated, the purpose in life item 7 (“I live life one day at a time and do not really think about the future”) had a low relationship with the latent trait. Therefore, this item must be revised and either modified or deleted.

## Discussion

A better understanding of psychological wellbeing is important because both flourishing and resilience are needed in order to survive, adapt, and thrive when facing the challenges of the 21^st^ century (Cloninger, 2004, 2013). In this context, we have argued that the measurement of psychological wellbeing is one cornerstone for identifying and treating both mental illness and health promotion. Measuring psychometric properties of the Swedish version of Ryff’s Psychological Wellbeing Scale is essential to make wellbeing assessment accessible and relevant to the Swedish-speaking population. Validating the scale in a new cultural context allows researchers and practitioners to better understand wellbeing within the Swedish culture, which may have unique aspects not captured by the original version. Moreover, culturally adapted scales enhance cross-cultural research and facilitate international comparisons, enriching our understanding of wellbeing across diverse populations. Through rigorous validation and cultural adaptation, the Swedish version of the scale can become a valuable tool for assessing and promoting wellbeing in the Swedish population, contributing to both research and practical applications in the field of psychology and public health. Hence, our aim was to explore and describe the psychometric properties of the 18-item Swedish version of Ryff’s Psychological Wellbeing Scale (Garcia, 2006) using both CTT and IRT methodologies. As far as we know, the present study is among the first to apply modern measurement theories, such as IRT, on this scale to address its psychometric properties.

Our results demonstrated that, as in previous studies (e.g., Lindfors et al., 2006; Van Dierendonck et al., 2008; Chan et al., 2017), a six-factor oblique model provided a relatively good fit for the 18-item Swedish version. However, the five-factor model, without the purpose in life subscale, had an even better fit. Importantly, the majority of past research has used the traditional linear factor model to examine the dimensionality of the scales, which is based on the assumption that responses are continuous scores on an interval scale metric (Ryff and Keyes, 1995; Van Dierendonck et al., 2008). Indeed, treating Likert scales as interval has been controversial for a long time (Knapp, 1990), but research indicates that they are fundamentally ordinal in nature (Norman, 2010; Wu and Leung, 2017). Therefore, we used Weighted Least Square (WLS) as the estimation method, since the WLS method provides more accurate and less biased results for ordinal data. More specifically, while Maximum Likelihood yields precise results for continuous and normally distributed data, diagonally WLS yields more accurate parameter estimates and model fit for ordinal and non-normal data (Beauducel and Herzberg, 2006; Mindrila, 2010; Koğar and Koğar, 2015; Li, 2016)—here the data was, for instance, not normally distributed.

Furthermore, the lack of discriminate validity of the theory-guided six-factor model may be attributable to the poor internal consistency of the purpose in life’s items and the small loadings of these items. That is, suggesting problems due to methodological features, such as design or wording of the items, negatively worded items, and semantic problems in the Sweden version of the 18-item version of Ryff’s Psychological Wellbeing Scale—as it is with other translated versions (Marsh, 1986; Mook et al., 1991; Cheng and Chan, 2005). For instance, the purpose in life item 7 (“I live life one day at a time and do not really think about the future”) might have been interpreted as positive, because it mirrors a way of living in the moment or “*Carpe Diem*.” This kind of approach to life has sometimes been seen as part of the good life by participants in different studies (*cf.* Tseferidi, Tseferidi et al., 2016), which might explain the negative loadings of this item. In addition, as also found by others (Clarke et al., 2001; Hsu et al., 2017), the lack of discrimination was accounted for by problematic items within three dimensions: environmental mastery, positive relations with others, and purpose in life. In this context, longer versions of Ryff’s Psychological Wellbeing Scale show better Cronbach’s alpha coefficients than shorter versions, while factorial validity fit indices are higher for shorter versus longer versions (Ryff and Singer, 2006). For instance, the psychometric investigation of multi-samples (Springer et al., 2006; Springer and Hauser, 2006), suggested that the 18-item version’s items may measure less than six dimensions (for a different opinion, see Ryff and Singer, 2006). In other words, the issues of a six-dimensional model versus other models, methodological problems, and *etcetera* seems to still be up for debate (see also Abbott et al., 2006).

One of our novel findings is the consistent five-factor structure of the 18-item Swedish version of Ryff’s scale across gender. This result contrasts with previous studies that reported gender differences in wellbeing factor patterns (Lindfors et al., 2006; Ryff and Singer, 2006; Chraif and Dumitru, 2015). Our finding indicates that the scale can equally measure psychological wellbeing in both men and women from the Swedish general population, suggesting gender universality in the wellbeing dimensions assessed by this scale. More specifically, for the 18-item version, distinctive gender patterns have been established for the positive relation with others subscale—with women reporting higher ability to create deep and meaningful connections with their others (Keyes et al., 2002; Lindfors et al., 2006; Matud et al., 2019). Moreover, in their study among 1,260 Swedish adults, Lindfors et al. (2006) found gender differences in purpose in life, environmental mastery, and positive relations with others. Nevertheless, our gender results match those in other studies that demonstrated that the factor structure of the 18-item version does not vary across gender (Linley et al., 2009). These results, however, need to be replicated.

Referring to the reliability of the 18-item Swedish version developed by Garcia (2006), our results were consistent with those regarding the 18-item version developed by Lindfors et al. (2006) that showed *Cronbach’s alpha* coefficients ranging from 0.24 for the purpose in life subscale to 0.70 for the self-acceptance subscale. In this study, the highest *Cronbach’s alpha* coefficient was also that of the self-acceptance subscale (0.77). However, both the autonomy (*α* = 0.49) and purpose in life subscales (*α* = 0.09) had unacceptable reliability (*cf.* George & Mallery, 2003). More specifically, in the purpose in life subscale, two out of the three items (i.e., item 7: “I live life one day at a time and do not really think about the future” and item 2: “I sometimes feel as if I’ve done all there is to do in life”) had very low relationships with both the subscale score and the total psychological wellbeing score. Thus, as discussed earlier, both items might have semantical issues that are problematic for our understanding of purpose in life as a construct of psychological wellbeing. That being said, several methodological problems such as reduced *Cronbach’s* alpha have been observed in previous studies as well (Keyes et al., 2002; Van Dierendonck, 2004; Lindfors et al., 2006; Sirigatti et al., 2009, 2013). For instance, Fernandes et al. (2010) study indicated that even after a set of re-specifications, *Cronbach’s* alpha values were low for all subscales (ranging from 0.27 to 50)—the purpose in life subscale being one of the two subscales with the lowest alphas (0.37 in Study 1 and 0.33 in Study 2). One possible reason is that the number of items is small for each subscale, but also that the items in this version were selected according to the conceptual and theoretical structure instead of the overvaluation of the psychometric criteria for internal consistency (Ryff and Keyes, 1995). However, in the current study, *Cronbach’s* alpha of 0.79 for the total psychological wellbeing score of the 18-item version confirms acceptable reliability, as suggested by Garcia and Siddiqui (2009). In addition, the inter-correlations were moderate between the positive relations with others subscale and both the environmental mastery and self-acceptance subscales and between the self-acceptance and environmental mastery subscales, while the other correlations were weak—besides the negative and near-to-zero correlation between purpose in life and autonomy. Hence, as suggested by Ryff (1989) and as shown in previous research (Ryff and Singer, 2006; Hsu et al., 2017), the subscales measure independent constructs.

Regarding the concurrent validity of the 18-item Swedish version of Ryff’s Psychological Wellbeing Scale, we found strong relationships between the subscales scores and the total psychological wellbeing score with the subjective wellbeing measures (i.e., positive affect, negative affect, and temporal satisfaction with life). Our study highlighted the relevance of subjective wellbeing measures in the context of psychological wellbeing. The strong relationships between the subscale scores and the total psychological wellbeing score with subjective wellbeing measures (positive affect, negative affect, and temporal satisfaction with life) reaffirm the interconnections between psychological wellbeing and subjective well-being concepts (Ryan and Deci, 2000; Fredrickson, 2004; Linley et al., 2009; Chen et al., 2013; Eguiarte and Miranda, 2016; Joshanloo, 2019). This finding underscores the importance of considering both objective and subjective aspects of wellbeing when developing comprehensive wellbeing promotion strategies. Additionally, discriminant validity was also demonstrated by the strong negative relationships between psychological wellbeing and health-related issues (i.e., both pain and sleeping problems), which is also in line with previous studies (Topcu, 2018; Zhai et al., 2018; see also Stålnacke, 2011; Ness and Saksvik-Lehouillier, 2018).

Furthermore, our results indicated that higher level of exercise frequency was positively related to the total score of psychological wellbeing and all subscales, except for autonomy and personal growth. Exercise intensity was positively associated with the total score of psychological wellbeing and all subscales, except for autonomy. Likewise, smoking had a negative relationship with the total score of psychological wellbeing and all subscales, except for autonomy. These results are also in line with previous studies (e.g., Norris et al., 1992; Brook et al., 2011; Garcia et al., 2012). Thus, our study provides additional evidence that the six psychological wellbeing dimensions, as measured by the 18-item version, are related to subjective wellbeing components, health, and lifestyle. However, as in most studies, we only assessed the link between self-reported measures of wellbeing, health, and lifestyle, thereby probably inflating the overall pattern of association. Thus, it would be useful for future studies to examine psychological wellbeing’s relations to other sociodemographic and biological factors.

Last but not least, the IRT analysis indicated that while the self-acceptance subscale offered the highest information, the purpose in life subscale offered the lowest. Therefore, it is plausible to suggest that all subscales but self-acceptance, should be revised to provide higher information values. Then, referring to the items, the IRT also showed that items 3, 4, 5, 7, 9, 10, 11, 13, 15, and 17 had low information and therefore, they need to be revised in order to enhance the psychometric properties and overall functioning of the scale. Moreover, given the very low discriminatory ability of item 2 as well as the negative slope value of item 7, our IRT analyses suggested the removal of these purpose in life items. In this regard, the results of the IRT analyses are consistent with those of the CTT. Thus, suggesting a five-factor structure (i.e., without the purpose in life subscale) for the 18-item Swedish version of Ryff’s Psychological Wellbeing Scale. Nevertheless, instead of deleting the items, we recommend exchanging them for more reliable and valid items. After all, both at the theoretical and empirical level having purpose in life is definitely part of human wellbeing (Ryff, 1989; Cloninger, 2004). Fortunately, Ryff’s (1989) original version contains 20 items per subscale and should therefore act as a perfect pool of items in this endeavor.

Assessing psychological wellbeing is pivotal for addressing mental health issues and promoting overall public health. Our study’s rigorous validation and adaptation of Ryff’s Psychological Wellbeing Scale offer a valuable tool for researchers, mental health practitioners, and policymakers in Sweden. The availability of a culturally adapted and psychometrically sound scale will enable more accurate and contextually relevant assessments of wellbeing in the Swedish population. From an economic perspective, understanding psychological wellbeing can have significant implications for workplace productivity and overall societal wellbeing. Organizations can utilize validated wellbeing measures to assess employee wellbeing and design targeted interventions to enhance workplace satisfaction and productivity. Additionally, health policymakers can use the scale to monitor and address mental health issues at the population level, leading to improved mental health outcomes and reduced healthcare costs.

### Limitations and future directions

The present study had several limitations. Firstly, the sample was obtained using volunteer sampling method and it is therefore not a representative sample of the Swedish population. Secondly, we used solely self-report instruments; therefore, the association between the variables might have been distorted by shared method variance. Further research needs to be conducted to assess its psychometric properties in various populations. Future research should, for example, examine the cross-cultural replicability of the scale’s factor structure through assessing samples from the indigenous populations in various countries and indigenous-language translations of the scale. Also, future longitudinal research is needed to test reciprocal and bidirectional associations between psychological wellbeing and other variables, such as personality. Future research should also include multi-informant assessments to provide a more conservative and more accurate test of the psychometric features of the 18-item version. Finally, given the contradictory results regarding gender differences in several studies, more research is needed to assess the 18-item version’s dimensionality among men and women.

## Conclusion

The 18-item version of Ryff’s Psychological Wellbeing Scale is an empirically supported tool for assessing psychological wellbeing based on the theoretical structure of Ryff’s multidimensional wellbeing model. This scale is widely used in research within the scope of positive mental health and several other contexts (e.g., education). To the best of our knowledge, this study is among the first to apply IRT to test the psychometric properties of the 18-item version of Ryff’s Psychological Wellbeing Scale and to provide new evidence regarding its factor structure in a Swedish sample. Our psychometric analyses did not support the original six-factor structure. Alternatively, a five-factor model without the purpose in life subscale was more adequate to assess psychological wellbeing with this Swedish version. Our results did, however, confirm satisfactory reliability and validity for the Swedish version of this scale in a general population. Taken all together, future studies should focus on modifying or exchanging items with low discrimination and information values and then examine whether the new scale improves the assessment of the six psychological wellbeing dimensions. Again, Ryff’s (1989) original version contains 20 items per subscale and should therefore act as a perfect pool of items in this endeavor. Hence, with regard to the current 18-item version, although we agree with Ryff and Singer (2006), who strongly encourage researchers to use all six dimensions when measuring psychological wellbeing; researchers should be cautious when interpreting the scores measured by the purpose in life factor and its related items (*cf.* Hsu et al., 2017)— Garcia, for example, recommended that the total score is a better and more reliable measure of psychological wellbeing when the 18-item Swedish version is used (Garcia and Siddiqui, 2009). At the end of the day, purpose in life as well as the other psychological wellbeing dimensions, are indeed some of the traits that the Science of Wellbeing (Cloninger, 2004, 2013) suggests as necessary for us humans to survive, adapt, and thrive during the challenges of the 21st century.

*“Instead of the usual outlook of separateness that leads to fear, excessive desire, and false pride, we can approach life with a self-transcendent outlook of unity that leads to love, hope, and humility functioning to serve others, not only ourselves. In this way, we can become both self-sufficient producers and moderate consumers. In other words, we can live sustainably with respect for our necessary harmony with nature and with the generosity needed to help others in a mutually beneficial way. Individual well-being is always a transient illusion when it is not coupled with collective well-being.”*

Cloninger (2013, p. 5)

## Data availability statement

The data analyzed in this study is subject to the following licenses/restrictions: the raw data supporting the conclusions of this article will be made available by the authors, without undue reservation. Requests to access these datasets should be directed to danilo.garcia@icloud.com.

## Ethics statement

Ethical approval and written informed consent were not required for the current study in accordance with the local legislation and institutional requirements. Ethical approval was not required for the studies involving humans because the present data was previously published open access (Garcia et al., 2016c). In the original study, after consulting with the Network for Empowerment and Well-Being’s Review Board, we arrived at the conclusion that the design of the present study (e.g., all participants’ data were anonymous and will not be used for commercial or other non-scientific purposes) required only informed consent from the participants. The studies were conducted in accordance with the local legislation and institutional requirements.

## Author contributions

DG wrote the manuscript and revised the manuscript as first author, designed the study, supervised the data collection, conducted statistical analysis, and interpreted the data. MHA wrote the manuscript and revised the manuscript equally as the first author, conducted statistical analysis, and interpreted the data. MK wrote the manuscript, conducted statistical analysis, interpreted the data, and revised the manuscript. All authors contributed to the article and approved the submitted version.
