# Corrigendum: The 18-item Swedish version of Ryff's psychological wellbeing scale: psychometric properties based on classical test theory and item response theory

**DOI:** 10.3389/fpsyg.2023.1324006

**Published:** 2023-11-03

**Authors:** Danilo Garcia, Maryam Kazemitabar, Mojtaba Habibi Asgarabad

**Affiliations:** ^1^Department of Behavioral Sciences and Learning, Linköping University, Linköping, Sweden; ^2^Centre for Ethics, Law and Mental Health (CELAM), University of Gothenburg, Gothenburg, Sweden; ^3^Promotion of Health and Innovation (PHI) Lab, International Network for Well-Being, Linköping, Sweden; ^4^Department of Psychology, University of Gothenburg, Gothenburg, Sweden; ^5^Department of Psychology, Lund University, Lund, Sweden; ^6^Yale School of Medicine, Yale University, New Haven, CT, United States; ^7^VA Connecticut Healthcare System, West Haven, CT, United States; ^8^Promotion of Health and Innovation (PHI) Lab, International Network for Well-Being, New Haven, CT, United States; ^9^Health Promotion Research Center, Iran University of Medical Sciences, Tehran, Iran; ^10^Department of Health Psychology, School of Behavioral Sciences and Mental Health (Tehran Institute of Psychiatry), Iran University of Medical Sciences, Tehran, Iran; ^11^Department of Psychology, Norwegian University of Science and Technology, Trondheim, Norway; ^12^Positive Youth Development Lab, Human Development and Family Sciences, Texas Tech University, Lubbock, TX, United States; ^13^Center of Excellence in Cognitive Neuropsychology, Institute for Cognitive and Brain Sciences, Shahid Beheshti University, Tehran, Iran

**Keywords:** classical test theory, item response theory, psychological wellbeing, psychometrics, wellbeing, health

In the published article, there was an error in Table 4 as published. The incorrect version of Table 4 was published. The corrected Table 4 and its caption appear below.

**Table 4:** Goodness-of-fit indices for confirmatory factor analysis of the 18-item Swedish version of Ryff's psychological wellbeing scales.

**Model**	**NNFI**	**AGFA**	**GFI**	**ECVI**	**CAIC**	**RMSEA**	**CFI**	**S-B χ^2^(df)**	**χ^2^/df**	**ΔS-B χ^2^(Δdf)**
M_0_	0.72	0.91	0.93	1.63	1,538.64	0.106 (0.10–0.11)	0.78	1,148.81 (120)	9.57	
M_1_	0.79	0.93	0.94	1.42	1,293.36	0.092 (0.087–0.097)	0.81	1,018.18 (135)	7.54	130.63 (15)^**^
M_2_	0.80	0.93	0.94	1.35	1,249.72	0.090 (0.084–0.095)	0.83	966.90 (134)	7.21	181.91 (14)^**^
M_3_	0.39	0.82	0.86	3.98	3,256.63	0.16 (0.15–0.16)	0.40	2,684.58 (135)	19.88	1,535.77 (15)^**^
M_4_	0.85	0.95	0.96	1.00	1,056.59	0.077 (0.071–0.083)	0.89	666.76 (120)	5.55	482.05^**^
M_5_	0.52	0.83	0.87	0.87	879.86	0.067 (0.062–0.073)	0.58	597.04 (129)	4.62	551.77 (9)^**^
M_6_	0.23	0.76	0.81	1.24	1,150.13	0.085 (0.079–0.090)	0.32	882.60 (136)	6.48	266.21 (16^)**^
M_7_	0.90	0.97	0.98	0.559	654.51	0.066 (0.059–0.073)	0.92	348.76 (80)	4.35	800.05 (40)^**^

M_0_, model with random item-factor associations; M_1_, general one-factor for 18 items; M_2_, two-factor orthogonal model; M_3_, six-factor orthogonal model; M_4_, six-factor oblique model; M_5_, six-factor first-order and second order model; M_6_, a six-factor first-order model and four-factor second-order that loaded by a four-factor model based on the four most highly correlated dimensions: environmental mastery, personal growth, purpose in life and self-acceptance; M_7_, five-factor first-order oblique model after removing the purpose in life subscale. ^**^ < 0.001.

The authors apologize for this error and state that this does not change the scientific conclusions of the article in any way. The original article has been updated.

